# Interface‐Mediated Catalysis for Improved Hydrogen Storage in Reactive Hydride Composites

**DOI:** 10.1002/adma.75169

**Published:** 2026-09-25

**Authors:** Yuanyuan Shang, Pawan Kumar Soni, Han Ge, Weijie Yang, Fahim Karimi, Thi Thu Le, Tamás Simon, Michael Fröba, Jitendra Mata, Joshua King, Hai‐Wen Li, Thomas Klassen, Claudio Pistidda

**Affiliations:** ^1^ Materials Design Institute of Hydrogen Technology Helmholtz‐Zentrum Hereon Geesthacht Germany; ^2^ Department of Physics School of Sciences IILM University Greater Noida India; ^3^ Institute of Inorganic and Applied Chemistry University of Hamburg Hamburg Germany; ^4^ Jinshuitan Hydropower Plant of State Grid Zhejiang Electric Power Co., Ltd China; ^5^ School of Energy and Power Engineering North China Electric Power University Baoding Hebei China; ^6^ The Hamburg Centre for Ultrafast Imaging Hamburg Germany; ^7^ Australian Centre for Neutron Scattering, ANSTO NSW Australia; ^8^ School of Chemistry University of New South Wales Sydney Australia; ^9^ Faculty of Science & Technology Charles Darwin University (CDU) Darwin NT Australia; ^10^ School of Advanced Energy Sun Yat‐sen University Shenzhen China; ^11^ Helmut Schmidt University Hamburg Germany

**Keywords:** hydrogen storage materials, interfaces, nanoparticles, reactive hydride composites

## Abstract

Improving the hydrogen storage properties of reactive hydride composite (RHC) systems remains challenging due to their high reaction barriers and multi‐step transformation pathways. Here, we report markedly enhanced hydrogen storage properties by incorporating Co@C and Ni@C catalysts into 2LiH + MgB_2_ system via an interface‐driven catalytic mechanism. Kinetic measurements exhibit significantly accelerated hydrogenation/dehydrogenation processes, accompanied by lower apparent activation energies. Temperature‐dependent investigations and synchrotron radiation powder x‐ray diffraction studies demonstrate a two‐step dehydrogenation process, in which the catalytic effect is mainly associated with LiBH_4_ decomposition. Multiscale structural characterization shows that the catalysts refine the hydride matrix and provide stable nanoscale interfaces that are maintained during cycling. Density functional theory calculations further reveal that the strong interfacial interactions between transition metal sites and LiBH_4_ lead to charge transfer and distortion of BH_4_^−^ units. The catalyst–hydride interfaces increase active site density, facilitate mass transport, and induce electronic interactions that weaken the B─H bonds and lower the dehydrogenation energy barrier. These findings demonstrate that the improved hydrogen storage properties predominantly result from interface‐driven processes rather than uniform bulk activation. This work provides a mechanistic framework for catalyst design through interfacial engineering, offering a general strategy for optimizing reactive hydride composites for energy storage applications.

## Graphical abstract

Catalyst–hydride interfaces are engineered in Co@C‐ and Ni@C‐modified 2LiH + MgB_2_ reactive hydride composites to accelerate hydrogen uptake and release. Multiscale scattering, in‐situ synchrotron radiation powder x‐ray diffraction and density functional theory calculations reveal the structural and electronic origin of the catalytic enhancement. Catalyst–hydride interfaces increase active site density, facilitate mass transport, weaken B─H bonds and reduce dehydrogenation barriers.

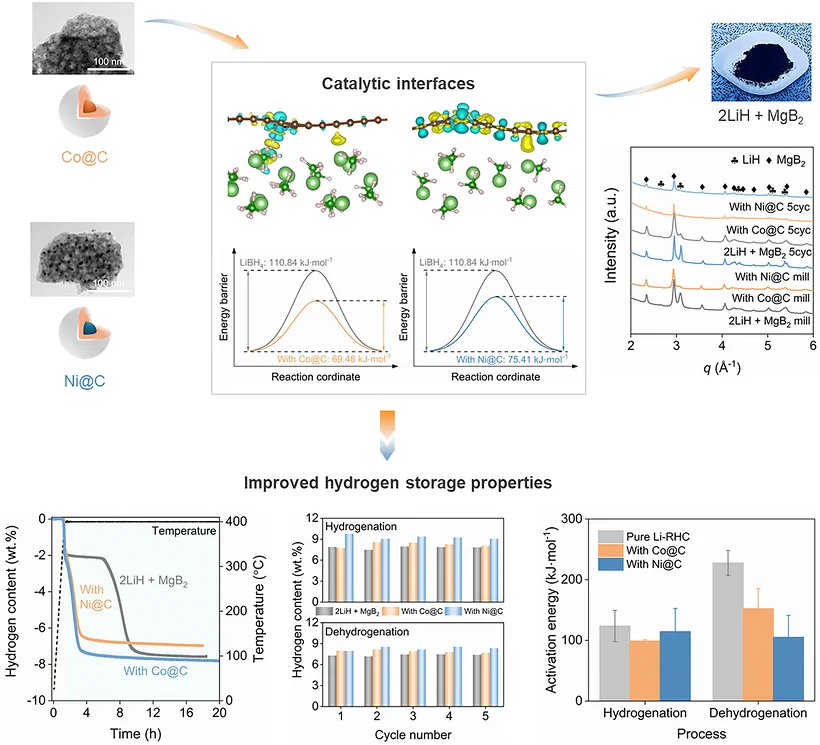

## 1 Introduction

With the world facing the dual challenges of climate change and energy security, there is a growing need to move away from fossil fuels and toward renewable energy sources. Hydrogen is increasingly being recognized as a crucial energy carrier of the transition to a sustainable, low‐carbon energy system, owing to its high gravimetric energy density (≈120 MJ kg^−1^) [1, 2]. Hydrogen storage is essential for the practical implementation of hydrogen energy, as it enables the transportation and distribution of hydrogen to end‐users. Among the different ways for storing hydrogen, material‐based solid‐state storage has the potential to significantly improve the practicality and safety of hydrogen energy systems [3, 4]. Metal hydrides are among the most used materials for solid‐state hydrogen storage because of their simple synthesis and strong potential for large‐scale applications [5, 6].

In recent years, Mg‐based reactive hydride composites (RHCs) have attracted significant attention as potential candidates for hydrogen storage because of their high gravimetric hydrogen density and tunable reaction enthalpy [7, 8]. RHCs typically consist of mixtures of different hydride species, such as simple hydrides and complex hydrides of alkali or alkaline earth metals, which can interact synergistically during the hydrogenation and dehydrogenation processes. As an example of this material class, the 2LiBH_4_ + MgH_2_ RHC possesses a theoretical hydrogen storage capacity of 11.5 wt.% (7.6 wt.% for MgH_2_) and a reaction enthalpy of 45.9 kJ mol^−1^ H_2_ (75.9 kJ mol^−1^ H_2_ for MgH_2_), as shown in the equations below [9, 10, 11]:

(1a) $$2\mathrm{LiB}H_{4}\left(s\right)+\mathrm{Mg}H_{2}\left(s\right)\to 2\mathrm{LiB}H_{4}\left(l\right)+\mathrm{Mg}\left(s\right)+H_{2}\left(g\right)$$

(1b) $$2\mathrm{LiB}H_{4}\left(l\right)+\mathrm{Mg}\left(s\right)\to 2\mathrm{LiH}\left(s\right)+\mathrm{Mg}B_{2}\left(s\right)+3H_{2}\left(g\right)$$

However, the still high thermodynamic stability, large kinetic barriers, and sluggish reaction kinetics hinder their practical application. To overcome these limitations, substantial research efforts have focused on enhancing the thermodynamic and kinetic properties of Mg‐based RHCs, particularly through the introduction of transition‐metal and metal oxide additives, carbon‐based materials, as well as nanostructuring strategies [12, 13, 14, 15]. For instance, Ding et al. synthesized nano 2LiBH_4_ + MgH_2_ mixture via a new processing method, referred to as ball milling with aerosol spraying (BMAS) [16]. The synthesized RHC can reversibly release 5.0 wt.% H_2_ at 265°C. In addition, additive‐based catalytic strategies in complex hydride systems can effectively shorten nucleation barriers. Transition‐metal‐based additives such as ZrCl_4_ and TiCl_3_, can reduce the nucleation induction period of MgB_2_ from 20 h to nearly zero [17]. Interestingly, the introduction of TiS_2_ into the 2LiBH_4_ + MgH_2_ RHC markedly enhances the reaction kinetics [18]. This effect is attributed to the bridging role of S^2−^ anion, which facilitates Li‐ion migration while simultaneously destabilizing the LiBH_4_ structure, thereby promoting rapid hydrogen diffusion. As a result, the hydrogenation/dehydrogenation kinetics are significantly improved, and excellent cycling stability is achieved, with over 90% capacity retention even after 50 cycles.

While these approaches can effectively enhance hydrogen storage kinetics, most studies attribute the improvement to a general catalytic acceleration without clearly identifying the specific reaction steps being affected. As a result, the fundamental catalytic mechanism in multi‐component hydride systems remains insufficiently understood, particularly regarding how catalysts interact with different hydride species at the microscopic level. In this context, recent studies suggest that catalytic performance may be governed not only by the intrinsic activity of the catalyst, but also by the nature of the catalyst–hydride interface [19, 20, 21, 22]. Theoretical calculations have shown that the interfacial interactions can significantly influence local electronic structures, thereby modulating bond strengths and reaction pathways [23]. However, the role of such interfacial effects in controlling individual reaction steps within RHC systems has rarely been clarified.

Within this context, catalytic interfaces were introduced into the 2LiBH_4_ + MgH_2_/2LiH + MgB_2_ RHC by incorporating Co@C/Ni@C catalysts. These catalysts provide hierarchical carbon‐supported structures with highly dispersed transition‐metal sites, enabling the formation of abundant and stable nanoscale interfaces with the hydride matrix. By combining kinetic measurements, in‐situ synchrotron radiation powder x‐ray diffraction (SR‐PXD), ultra‐small‐angle and small‐angle neutron scattering (USANS & SANS), density functional theory (DFT) calculations and so on, we demonstrate that the catalytic enhancement originates from an interface‐driven activation mechanism. Specifically, the Co@C/Ni@C catalysts preferentially interact with LiBH_4_, inducing charge transfer and weakening B─H bonds, thereby lowering the energy barrier for its decomposition. This work provides new insight into the role of interfacial interactions in RHC systems and highlights a general strategy for optimizing hydrogen storage through interfacial design.

## 2 Results and Discussion

### 2.1 Synthesis and Structural Characteristics of Co@C/Ni@C Catalysts

The Co@C/Ni@C catalysts were constructed via a polymer‐assisted self‐assembly strategy, in which organic precursors and surfactant templates (Pluronic P123) guide the formation of a mesostructured polymer network, followed by carbonization and incorporation into the 2LiH + MgB_2_ system (Figure 1a). This approach enables the formation of mesoporous carbon frameworks with embedded transition metal species, providing a hierarchical structure with well‐dispersed active sites and promoting the formation of abundant catalyst–hydride interfaces. The surface morphology of the Co@C sample (Figure 1b) is comparatively uneven and rough, with loosely packed particles dispersed throughout the carbon matrix. This indicates the formation of the nanostructures of the Co, which is embedded in the porous carbon framework. The Ni@C sample (Figure 1e) has better particle connectivity and dispersion and a more homogeneous and compact shape. The rigid structure of Ni@C is further confirmed by the relatively uniform structure, indicating a more regular proliferation and dispersion of the Ni species during the carbonization. Importantly, the particle agglomeration of the samples and/or collapse in porosity structure is not significant, indicating that the carbon framework has remained robust after the insertion of the Co and Ni. The interconnected porous structure is beneficial for both materials as it provides a larger surface area and higher accessibility to active sites and can enhance the catalytic activity. The scanning electron microscopy (SEM) analysis has proven the synthesized well‐structured and particle‐distributed mesoporous carbon supported Co and Ni nanocomposites with good structural integrity. Further structural insights are provided by transmission electron microscopy (TEM) images (Figure 1c,f). They have also clearly confirmed the successful embedding of the Co and Ni nanoparticles inside the mesoporous carbon network. In the Co@C sample (Figure 1c), Co nanoparticles are uniformly distributed in the carbon matrix and are very fine. The particles are relatively small, and well enclosed in the porous carbon structure, which shows the formation of Co nanostructures was effective in the synthesis process. The surrounding carbon framework seems to be partially amorphous, a feature that is often seen in carbonized porous materials. The TEM image of the Ni@C sample (Figure 1f) shows clear dark contrast areas, which represent the Ni nanoparticles dispersed in the carbon material. In comparison with Co@C, the Ni nanoparticles look a bit larger, but more evenly dispersed throughout the carbon structure. This implies an enhanced interaction between the Ni species and the carbon precursor during carbonization, and thus an improved dispersion and stabilization of the particles. The particle size distribution analysis also agrees with the TEM images, the average particle sizes of Co@C are about 3.51 nm (Figure 1d) and of Ni@C are about 4.74 nm (Figure 1g). The fact that the two samples have a narrow particle size distribution suggests that the growth of the nanoparticles is controlled in the mesoporous carbon environment. Importantly, no significant nanoparticle agglomeration has been detected in either sample, suggesting that the carbon framework prevents particle agglomeration during the nanoparticles’ synthesis and heat treatment. The close contact of the metallic nanoparticles with the conductive carbon matrix would likely enhance the stability of the structure and allow efficient electron transport, beneficial for catalytic application. From a general point of view, the TEM analysis demonstrates the good synthesis of well‐dispersed Co and Ni nanoparticles in stable mesoporous carbon network.

**FIGURE 1:**
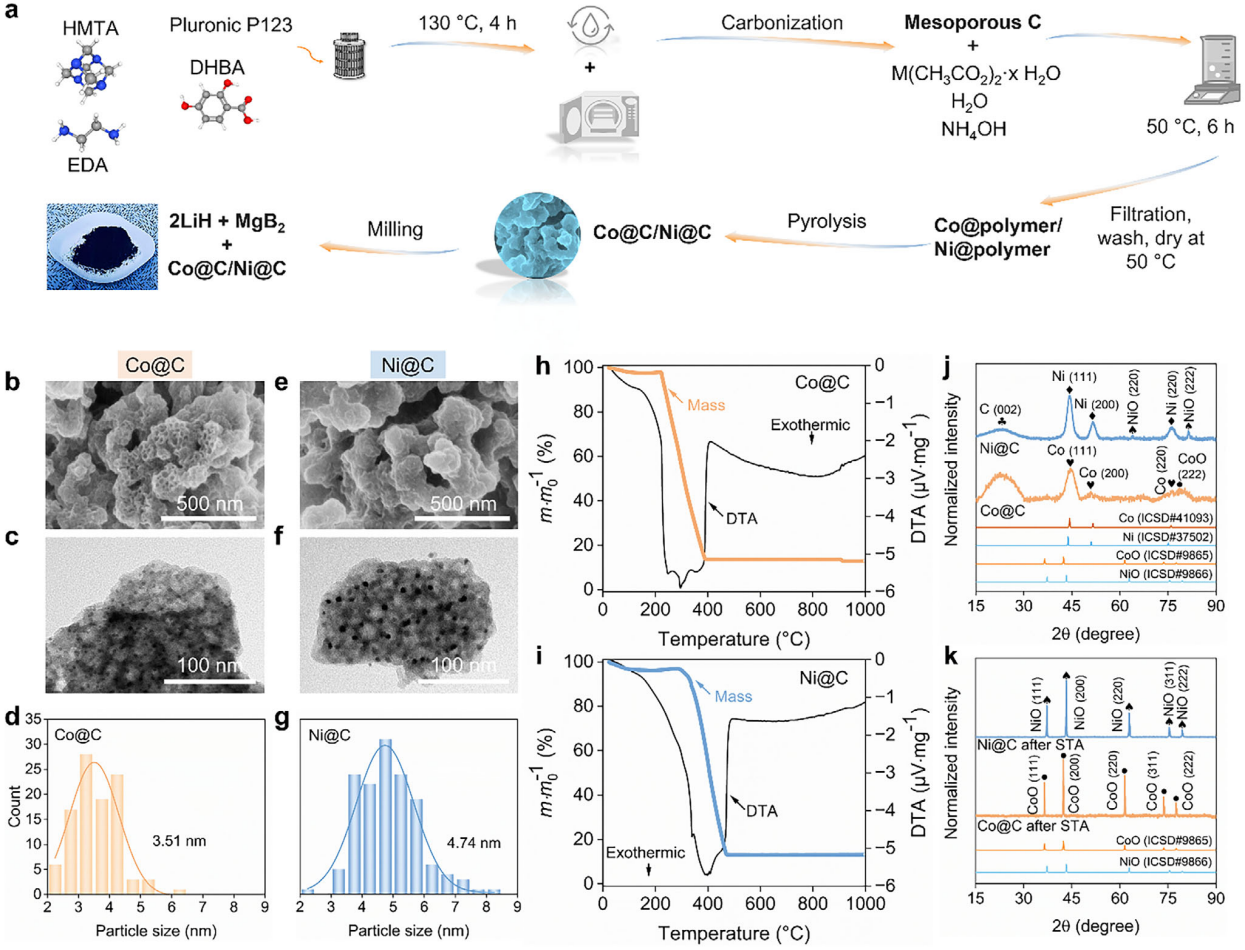
Synthesis of 2LiH + MgB_2_ + Co@C/Ni@C composites and structural characterization of the Co@C and Ni@C catalysts. (a) Schematic illustration of the synthesis route for 2LiH + MgB_2_ + Co@C/Ni@C composites. (b)–(d) Scanning electron microscopy (SEM) and transmission electron microscopy (TEM) images, as well as particle size distribution derived from TEM data of the Co@C catalyst. (e)–(g) SEM and TEM images, as well as particle size distribution derived from TEM data of the Ni@C catalyst. (h), (i) Thermogravimetric analysis and differential thermal analysis (TGA‐DTA) of the Co@C and Ni@C catalysts. (j), (k) XRD patterns of the two catalysts before and after TGA‐DTA measurement. The reference XRD patterns obtained from ICSD are included for comparison. Wavelength (*λ*) = 1.5418 Å.

Simultaneous thermal analysis and differential thermal analysis (STA–DTA) (Figure 1h,i) analysis can assist in the understanding of the decomposition of precursor materials, the metal content and the overall thermal stability of the final composites. The measurements were conducted from room temperature to 1000°C at a heating rate of 5°C min^−1^ under an Ar/O_2_ atmosphere (4:1, *v*/*v*). Both Co@C and Ni@C exhibit significant mass loss accompanied by exothermic features (Figure 1h,i), which can be attributed to the thermal decomposition of the carbon matrix and particle oxidation during heating. The final oxidation products contain 12.9 wt.% CoO and 13.2 wt.% NiO, corresponding to metallic Co and Ni contents of 10.1 wt.% and 10.4 wt.%, respectively. These results indicate comparable metal loadings in both catalysts, confirming the successful incorporation of transition metal species within the carbon matrices. The x‐ray diffraction (XRD) patterns clearly show that the crystalline metallic Co and Ni nanoparticles have been successfully encapsulated in the carbon frameworks. For Co@C, the (1 1 1), (2 0 0) and (2 2 0) planes of face‐centered cubic (*fcc*) Co (ICSD Collection Code: 41093) can be seen as individual diffraction peaks (Figure 1j), which show that crystalline Co nanoparticles are present. Likewise, the diffraction peaks could be identified as characteristic peaks (1 1 1), (2 0 0) and (2 2 0) of metallic *fcc* Ni (ICSD Collection Code: 37502) in the Ni@C sample (Figure 1j). Both samples showed characteristic relatively broad diffraction peaks indicating the presence of nanoparticles with nanoscale crystallite size, which is consistent with that determined by TEM. Also, both samples have a broad peak around 2*θ* ≈ 25°, which is due to the (0 0 2) plane of carbon. This is a wide feature and suggests that the carbon matrix is predominantly amorphous and there is some graphitic ordering that occurs during carbonization. After thermal treatment, the appearance of CoO (ICSD Collection Code: 9865) and NiO (ICSD Collection Code: 9866) indicates phase stabilization and potential oxidation, as shown in Figure 1k. The corresponding evolution of the diffraction features is in good agreement with the STA–DTA curves. Therefore, the XRD data confirm successful synthesis of Co and Ni nanoparticles uniformly embedded in the mesoporous carbon structure. Together, the combined microscopy, thermal and diffraction analyses demonstrate that both Co@C and Ni@C catalysts consist of finely dispersed metal nanoparticles confined within mesoporous carbon frameworks. Such structural characteristics are expected to play a crucial role in modulating the hydrogen storage behavior of the 2LiH+MgB_2_ RHC system.

### 2.2 Hydrogen Storage Behavior of the 2LiH + MgB_2_ + Co@C/Ni@C RHCs

The hydrogen storage behavior of the 2LiH + MgB_2_ system is significantly affected by the introduction of 7 wt.% Co@C/Ni@C catalysts. Note that all hydrogen storage capacities are reported relative to the total mass of the composite, including the 7 wt.% catalyst. As shown in Figure 2a, during the first cycle, the pristine 2LiH + MgB_2_ exhibits relatively sluggish hydrogenation and dehydrogenation kinetics. Here, *t*_90_ is defined as the time required to reach 90% of the final capacity attained in each hydrogenation/dehydrogenation measurement. The *t*_90_ values of pristine 2LiH + MgB_2_ are approximately 5.7 and 3.6 h for hydrogenation and dehydrogenation, respectively. Its final hydrogen storage capacities for hydrogenation and dehydrogenation are 7.89 and 7.31 wt.%, respectively. In comparison, the Co@C‐doped sample reaches *t*_90_ after 7.6 h during hydrogenation and 2.0 h during dehydrogenation, achieving final capacities of 7.74 and 8.00 wt.%, respectively. For the Ni@C‐doped sample, the *t*_90_ time for hydrogenation is 6.8 h, with a final hydrogenation capacity of 9.77 wt.%. Compared to the Co@C‐doped sample, the Ni@C‐doped sample shows further improved dehydrogenation kinetics, reaching *t*_90_ after 1.1 h during dehydrogenation, while delivering a final capacity of 7.95 wt.%. In contrast to the undoped composite, the samples containing Co@C and Ni@C require an additional 1.9 and 1.1 h, respectively, to achieve *t*_90_ during the first hydrogenation. Since *t*_90_ is determined relative to the sample's final capacity, it should be interpreted alongside the corresponding hydrogen uptake. According to the total composite mass, the Co@C sample demonstrates only a 0.15 wt.% reduction in capacity (approximately 1.9%), whereas the Ni@C sample reveals a 1.88 wt.% increase (approximately 23.8%). Given an estimated measurement uncertainty of 5%, differences of comparable magnitude should be treated with caution. Following normalization to the 93 wt.% RHC fractions, the capacities reach 8.32 and 10.51 wt.% for Co@C and Ni@C‐doped samples, respectively, in comparison with 7.89 wt.% for the undoped composite. Consequently, Co@C appears to compensate for the catalyst‐related reduction of system storage capacity, whereas Ni@C offers a clear enhancement in capacity that goes beyond the measurement uncertainty. More importantly, the dehydrogenation kinetics are significantly enhanced, which indicates pronounced improvements in the rate‐limiting steps, especially associated with the decomposition of LiBH_4_. A similar trend is observed in the fifth cycle (Figure 2b), indicating that the kinetic enhancement is well maintained upon cycling. The undoped 2LiH + MgB_2_ RHC reaches *t*_90_ after 2.8 h during hydrogenation and 3.4 h during dehydrogenation, respectively. In contrast, the corresponding values for the Co@C‐doped sample are 4.3 and 3.3 h, respectively. The Ni@C‐doped sample exhibits faster kinetics, reaching *t*_90_ after 2.3 and 2.5 h, respectively. Among the investigated samples, the Ni@C‐containing sample exhibits superior overall kinetic and storage properties. In contrast, the addition of Co@C does not reduce the hydrogenation *t*_90_ compared with the undoped composite. Nevertheless, its beneficial effects remain evident in the higher final capacity and the improved dehydrogenation behavior of the Co@C‐doped sample.

**FIGURE 2:**
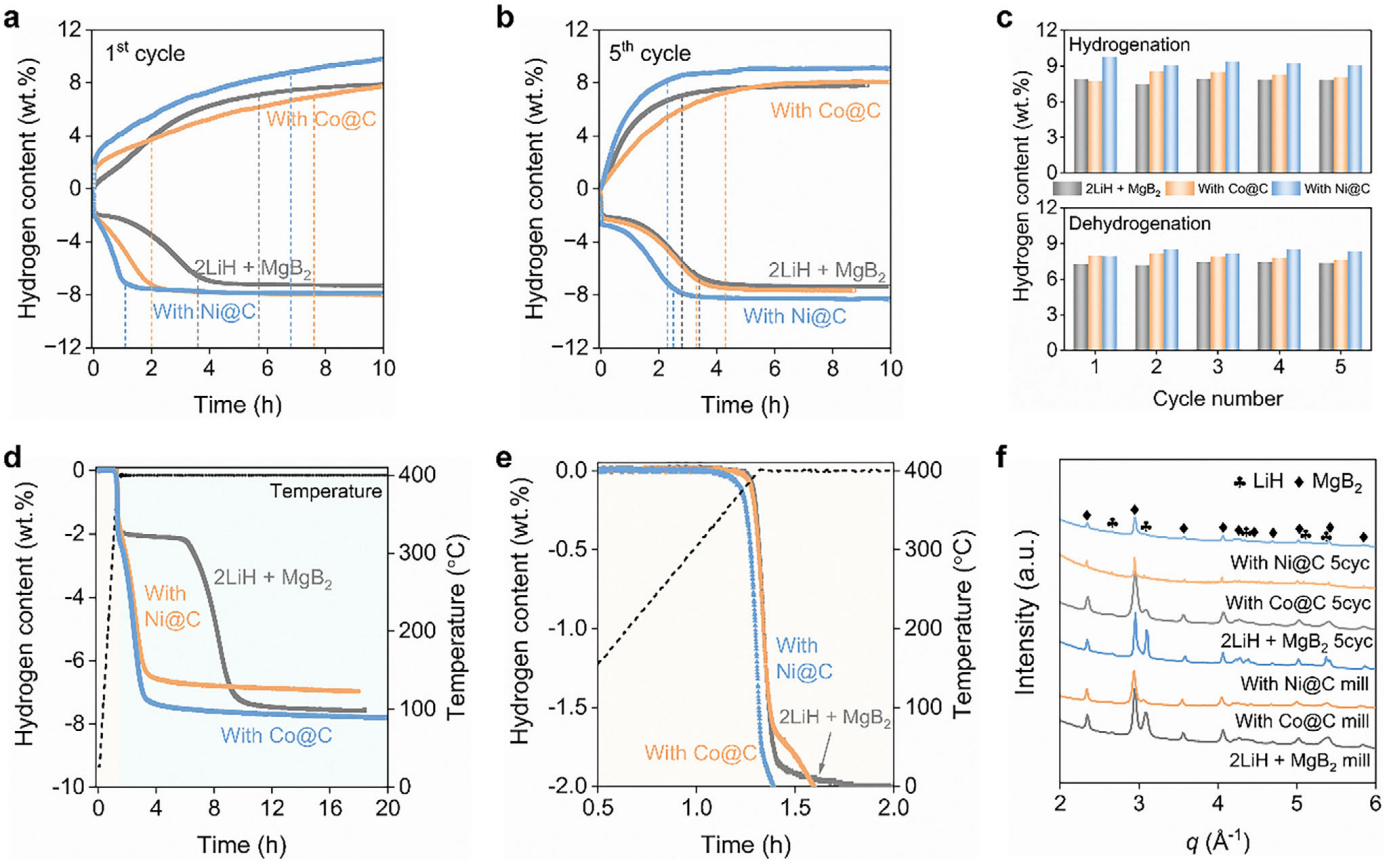
Hydrogen storage kinetics and synchrotron radiation powder x‐ray diffraction (SR‐PXD) analysis of 2LiH + MgB_2_ + Co@C/Ni@C composites. (a), (b) Hydrogenation/dehydrogenation behavior during the first and fifth cycles for undoped and Co@C/Ni@C doped 2LiH + MgB_2_ composites. (c) The overall hydrogenation/dehydrogenation capacities of the undoped and Co@C/Ni@C doped 2LiH + MgB_2_ composites over repeated cycles. (d), (e) Dehydrogenation curves of the hydrogenated undoped and Co@C/Ni@C doped 2LiH + MgB_2_ composites measured under 3 bar of hydrogen, from room temperature to 400°C at a heating rate of 10°C min^−1^, followed by keeping isothermal at 400°C. f SR‐PXD patterns of the undoped and Co@C/Ni@C doped 2LiH + MgB_2_ composites before and after five hydrogenation/dehydrogenation cycles. Note that all hydrogen storage capacities are reported relative to the total mass of the composite, including the 7 wt.% catalyst.

The cycling performance in Figure 2c further confirms that the Co@C/Ni@C‐doped composites exhibit stable hydrogenation and dehydrogenation capacities over repeated cycles. The detailed kinetic curves for all cycles can be found in Figure S1. Compared with the undoped 2LiH + MgB_2_ RHC, the doped samples possess consistently higher hydrogen storage capacities, with the Ni@C‐doped sample showing the most significant improvement. After five cycles, the undoped RHC delivers hydrogenation and dehydrogenation capacities of 7.84 and 7.40 wt.%, respectively. In comparison, the Co@C‐doped sample shows slightly improved values of 8.06 and 7.66 wt.%, while the Ni@C‐doped sample achieves significantly higher capacities of 9.06 and 8.35 wt.%, respectively. Note that the measured dehydrogenation capacities are slightly lower than the corresponding hydrogenation capacities. This discrepancy can be attributed to the rapid initial dehydrogenation of MgH_2_, which partially occurs beyond the detection limit of the Sieverts’ apparatus. Nevertheless, throughout cycling, the catalyzed systems maintain higher capacities than the undoped composite. For instance, the initial hydrogenation capacity of the Ni@C‐doped sample is 1.88 wt.% higher than that of the undoped RHC, and this improvement remains after five cycles (1.22 wt.% higher). The capacities remain almost constant with negligible degradation, indicating a stable cycling behavior of the catalyzed systems.

Temperature‐programmed dehydrogenation measurements (Figure 2d,e) reveal that the dehydrogenation process occurs in two distinct stages. The first stage is associated with the decomposition of MgH_2_, while the second stage corresponds to the decomposition of LiBH_4_. Clearly, the comparable onset and release characteristics in the first stage illustrated in Figure 2e indicate that the introduction of Co@C and Ni@C catalysts does not substantially influence the MgH_2_ decomposition behavior. In contrast, a pronounced acceleration is observed in the second stage, corresponding to LiBH_4_ decomposition. In the presence of Co@C and Ni@C catalysts, this process initiates much earlier (at 1.3 and 1.4 h, respectively), compared to 6.0 h for the undoped RHC. In addition, it proceeds at a notably faster rate. The distinct incubation period observed in the pristine 2LiH + MgB_2_ system disappears after the addition of catalysts, suggesting that Co@C/Ni@C can effectively lower the kinetic barrier related to the onset of LiBH_4_ decomposition. The reduced dehydrogenation duration is mainly attributed to the second reaction step, which is associated with accelerated LiBH_4_ decomposition, as elaborated upon in the subsequent structural and theoretical results. The phase constitutions before and after cycling were investigated using SR‐PXD (Figure 2f). All samples retain good reversibility upon cycling, as no detectable additional phases appear. This observation implies that the hydrogenation/dehydrogenation processes proceed without irreversible reactions, for example, formation of LiB_12_H_12_ phase, thereby preserving the system's structural integrity. Overall, the addition of Co@C/Ni@C catalysts can effectively enhance the hydrogen storage performance of the 2LiH + MgB_2_ RHC, primarily by accelerating the dehydrogenation processes of LiBH_4_, while still maintaining reversibility over multiple cycles.

### 2.3 Temperature‐Dependent Kinetics and Activation Energies

To further elucidate the catalytic effect of Co@C/Ni@C on the hydrogen storage performance, the hydrogenation and dehydrogenation kinetics of the undoped and doped 2LiH + MgB_2_ composites were systematically studied over a range of temperatures (Figure 3). For the undoped 2LiH + MgB_2_ RHC (Figure 3a), the hydrogenation process shows a clear temperature dependence. At 325°C, hydrogenation proceeds slowly and does not reach full capacity within the measured time, indicating limited kinetics. As the temperature increases to 350°C, 360°C, and 375°C, the hydrogenation rate progressively improves, resulting in faster hydrogen uptake and higher final capacities within shorter reaction times. Nevertheless, the overall hydrogenation process remains relatively sluggish even at elevated temperatures, indicating that the undoped system is intrinsically limited by slow reaction kinetics. A similar temperature‐dependent behavior is observed in the dehydrogenation process (Figure 3b). At lower temperature, that is, 375°C, hydrogen release is greatly delayed and proceeds slowly, whereas increasing the temperature to 400°C, 410°C, and 425°C accelerates the dehydrogenation process and shortens the time to reach equilibrium. However, the extended dehydrogenation time indicates that hydrogen release in the undoped RHC remains kinetically hindered in the absence of catalysts. With the introduction of Co@C (Figure 3d,e), the hydrogenation kinetics and capacities are markedly improved across the entire temperature range. Additionally, the dehydrogenation behavior is also greatly enhanced, with faster hydrogen release and reduced time for completion of the reaction. These findings show that the Co@C catalyst efficiently promotes the hydrogenation and dehydrogenation processes. The Ni@C‐doped system features a more noticeable enhancement (Figure 3g,h). The hydrogenation process proceeds quickly, reaching near‐equilibrium states within a short period. Even at a relatively low temperature of 325°C, a rapid hydrogenation process is observed, where the system approaches near‐equilibrium within a time that is four times shorter than that for the undoped RHC. Similarly, the dehydrogenation process is significantly accelerated, which dramatically reduces the reaction time. This trend implies that Ni@C offers more efficient catalytic sites for hydrogen‐related reactions.

**FIGURE 3:**
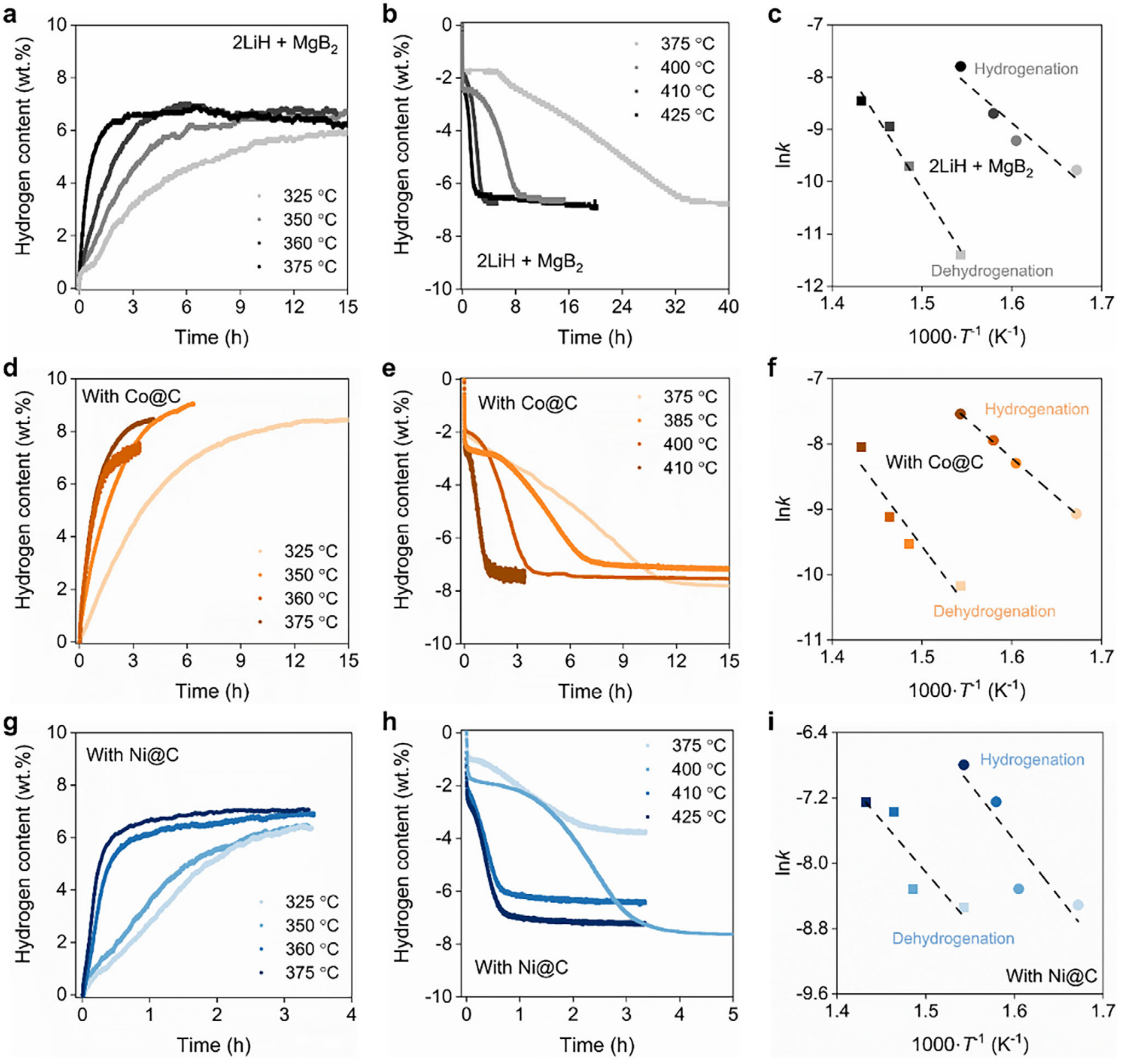
Hydrogen storage properties at different temperatures and the corresponding activation energies (*E*_a_) of 2LiH + MgB_2_ + Co@C/Ni@C composites. (a) Hydrogenation properties of 2LiH + MgB_2_ at 325°C, 350°C, 360°C and 375°C under 50 bar of hydrogen. (b) Dehydrogenation properties of hydrogenated 2LiH + MgB_2_ at 375°C, 400°C, 410°C, and 425°C under 3 bar of hydrogen. (c) Calculations of hydrogenation/dehydrogenation *E*_a_ based on the data in (a) and (b). (d) Hydrogenation properties of 2LiH + MgB_2_ + Co@C at 325°C, 350°C, 360°C, and 375°C under 50 bar of hydrogen. (e) Dehydrogenation properties of hydrogenated 2LiH + MgB_2_ + Co@C at 375°C, 385°C, 400°C, and 410°C under 3 bar of hydrogen. (f) Calculations of hydrogenation/dehydrogenation *E*_a_ based on the data in (d) and (e). (g)Hydrogenation properties of 2LiH + MgB_2_ + Ni@C at 325°C, 350°C, 360°C, and 375°C under 50 bar of hydrogen. (h) Dehydrogenation properties of hydrogenated 2LiH + MgB_2_ + Ni@C at 375°C, 400°C, 410°C, and 425°C under 3 bar of hydrogen. (i) Calculations of hydrogenation/dehydrogenation *E*_a_ based on the data in g and h.

The kinetic improvements are further quantified by the apparent activation energies (*E*_a_) determined from the Arrhenius plots (Figure 3c,f,i). The undoped RHC shows relatively high activation energies for both hydrogenation (*E*_a_ = 123.7 ± 25.7 kJ mol^−1^ H_2_) and dehydrogenation (*E*_a_ = 227.7 ± 20.5 kJ mol^−1^ H_2_) processes, indicating the intrinsic kinetic barriers of the system. Upon catalyst addition, the activation energies are markedly reduced, confirming that the catalysts effectively lower the energy barriers associated with hydrogenation and dehydrogenation. For the Co@C‐doped sample, the hydrogenation and dehydrogenation activation energies decrease to 99.1 ± 2.1 kJ mol^−1^ H_2_ and 152.3 ± 33.1 kJ mol^−1^ H_2_, respectively. In comparison, the Ni@C‐doped RHC exhibits a hydrogenation activation energy of 114.5 ± 38.2 kJ mol^−1^ H_2_ and a significantly lower dehydrogenation activation energy of 105.2 ± 36.0 kJ mol^−1^ H_2_. Notably, the reduction in activation energy is more significant for the dehydrogenation process than for hydrogenation, which is consistent with the accelerated dehydrogenation process observed in Figure 2d,e. This trend suggests that catalysts play a particularly important role in facilitating the dehydrogenation step. Combined with the two‐step dehydrogenation behavior discussed in Figure 2, this observation implies that the catalytic effect is closely related to the transformation of specific hydride species, mostly related to the LiBH_4_ decomposition step.

### 2.4 Phase Evolution and Reaction Pathway During Dehydrogenation

To elucidate the origin of the catalytic enhancement, the phase evolution during dehydrogenation was investigated using in‐situ SR‐PXD (Figure 4). For the Co@C‐doped system (Figure 4a,b), the contour plot and selected diffraction patterns reveal a sequential transformation of the initial phases during heating. Clearly, LiBH_4_ undergoes a structural phase transformation at ca. 155°C. When the temperature reaches 339°C, the LiBH_4_ is fully melted. Dehydrogenation of MgH_2_ occurs mainly during the isothermal stage at 425°C. Note that the formation of MgB_2_ is also observed, which is related to the decomposition of LiBH_4_. The formation of LiH is detected during cooling, at around 290°C. As the temperature increases, a similar phase evolution behavior is also observed in the Ni@C‐catalyzed system (Figure 4c,d). The structural phase transformation of LiBH_4_ happens at 155°C, which is in good agreement with the Co@C‐doped sample. The LiBH_4_‐related diffraction peaks diminish at 348°C, accompanied by the earlier formation of product phases. Then the peaks of LiH start to become visible when cooling to 382°C, which indicates that the faster dehydrogenation of LiBH_4_ compared to the Co@C‐doped sample. When the sample was subsequently cooled to 148°C, the peaks of MgB_2_ are more pronounced. These observations are consistent with the two‐step dehydrogenation behavior identified in Figure 2, confirming that the catalytic effect is predominantly associated with the LiBH_4_ decomposition step. The acceleration of LiBH_4_ transformation suggests that the catalysts interact preferentially with borohydride species, thereby facilitating the corresponding hydrogen release reactions.

**FIGURE 4:**
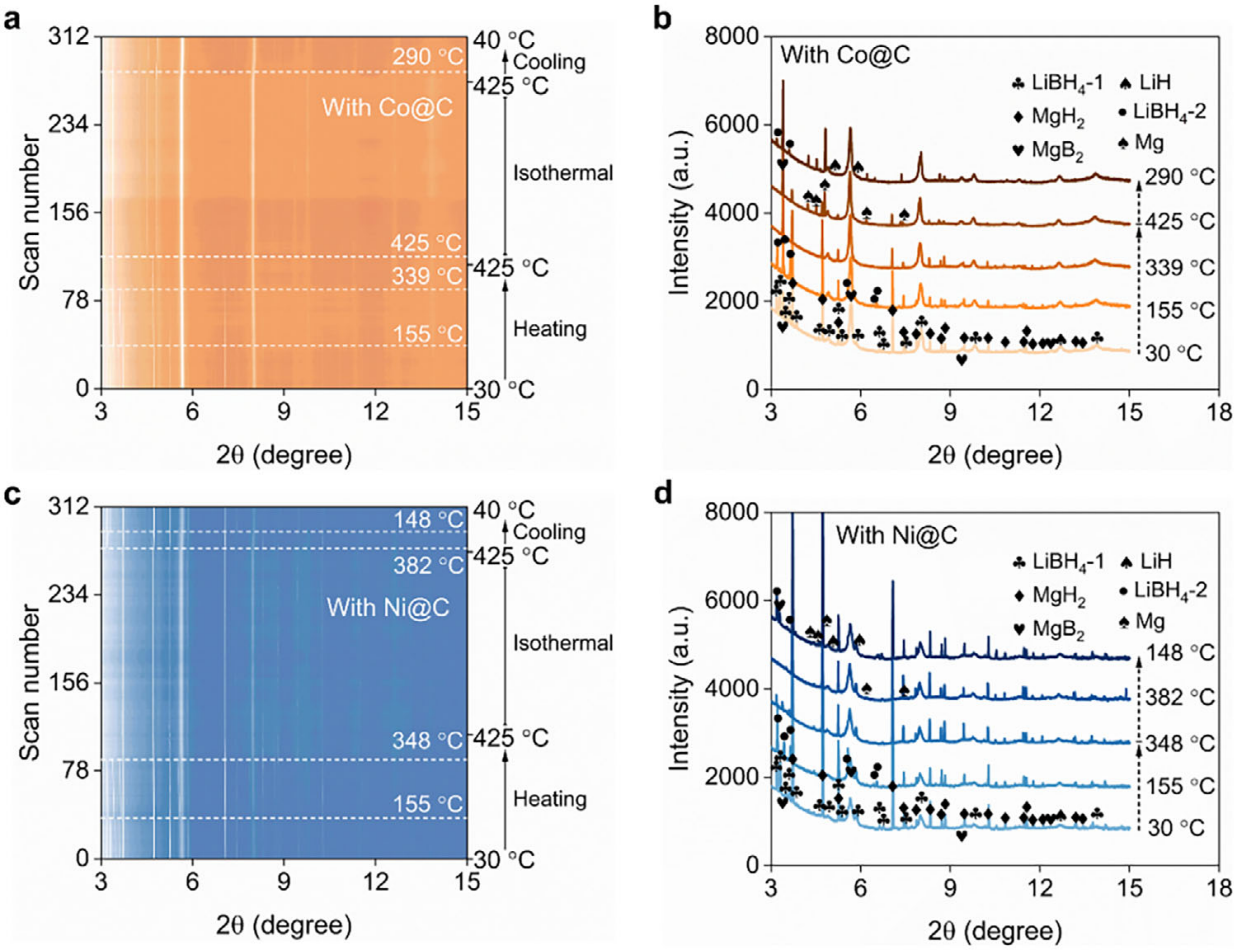
Dehydrogenation phase evolution of hydrogenated 2LiH + MgB_2_ + Co@C/Ni@C composites obtained by in‐situ SR‐PXD. (a) In‐situ SR‐PXD contour plot of the Co@C‐catalyzed composite during heating, isothermal holding, and cooling. (b) Selected SR‐PXD patterns from a at representative temperatures. (c) In‐situ SR‐PXD contour plot of the Ni@C‐catalyzed composite during heating, isothermal holding, and cooling. d Selected SR‐PXD patterns from (c) at representative temperatures. *λ* = 0.20739 Å.

### 2.5 Hierarchical Structural Features and Their Stability During Cycling

To further elucidate the structural basis of the catalytic effect, the multiscale morphology and its evolution during hydrogenation/dehydrogenation were investigated using USANS & SANS (Figure 5), which together probe structural features from nanometer to micrometer length scales. The scattering curves were fitted using spherical form factors as a first approximation. Given the complexity of the system, particle size distributions were analyzed using SASfit software with a log‐normal distribution model [24]. The fitted populations were assigned by comparing the characteristic size ranges obtained for the pristine RHC, isolated catalysts and catalyst‐containing composites. The smallest population is consistent with the metal‐containing nanoparticles observed by TEM, with typical radii of approximately 5–8 nm. The intermediate population is assigned to the RHC matrix since it is also present in the pristine composite. The largest population, as initially identified in the isolated catalysts, is assigned to the carbon scaffold. These assignments are inferred from the combined scattering and microscopy data rather than uniquely determined by the fitting alone. The fitting parameters obtained from the combined sphere‐model analysis of the USANS & SANS data can be found in Table S1. Note that all characteristic dimensions reported in Figure 5 are equivalent‐sphere radii. The scattering profiles of the Co@C and Ni@C catalysts (Figure 5b) can be well reproduced by the fitted curves, indicating hierarchical structures across multiple length scales. The corresponding size distributions (Figure 5c,d) reveal two characteristic structural populations. One is assigned to the carbon scaffolds with average radii of 155.7 and 170.6 nm for Co@C and Ni@C, respectively. The other is assigned to embedded nanoparticles with radii of only a few nanometers (6.6 and 5.8 nm, respectively). These findings are consistent with the fact that both catalysts comprise nanosized metal‐containing particles distributed within carbon frameworks. They can serve as a structural basis for extensive catalyst–hydride interfacial interactions after mixing with the 2LiH + MgB_2_ RHC.

**FIGURE 5:**
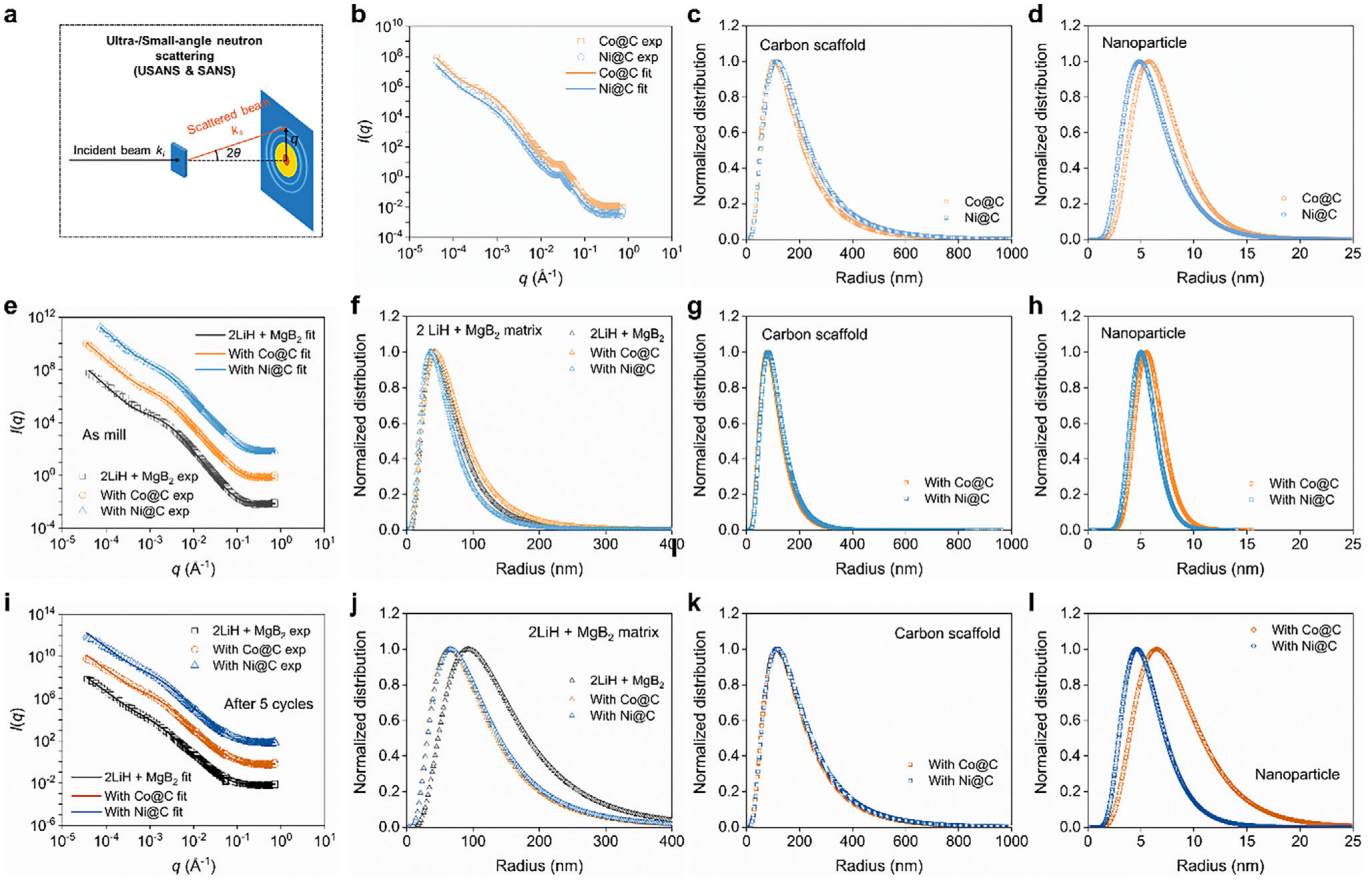
Ultra‐small‐angle and small‐angle neutron scattering (USANS & SANS) characterizations of the 2LiH + MgB_2_ + Co@C/Ni@C composites before and after hydrogenation/dehydrogenation cycles. (a) Schematic illustration of the USANS & SANS operating principle. (b) Experimental and fitted USANS & SANS profiles of Co@C and Ni@C catalysts. (c), (d) Fitted size distributions of carbon scaffolds and nanoparticles in Co@C and Ni@C catalysts. (e) Experimental and fitted USANS & SANS patterns of as‐milled 2LiH + MgB_2_ + Co@C/Ni@C composites. (f)–(h) Fitted size distributions of 2LiH + MgB_2_ matrix, carbon scaffolds and nano particles in as‐milled 2LiH + MgB_2_ + Co@C/Ni@C composites. (i) Experimental and fitted USANS & SANS patterns of 2LiH + MgB_2_ + Co@C/Ni@C composites after five hydrogenation/dehydrogenation cycles. (j)–(l) Fitted size distribution of 2LiH + MgB_2_ matrix, carbon scaffolds and nanoparticles in 2LiH + MgB_2_ + Co@C/Ni@C composites after five hydrogenation/dehydrogenation cycles.

In the as‐milled composites (Figure 5e), clear differences are observed between the pristine and catalyst‐containing systems. Both Co@C‐ and Ni@C‐doped composites exhibit noticeably different scattering profiles over a wide *q* range compared with the undoped 2LiH + MgB_2_ sample, suggesting that the addition of catalysts alters the hydride mixture's multiscale structural configuration. These variations are further confirmed by the size distributions extracted from the fitted curves (Figure 5f–h). The undoped sample shows a matrix distribution with an average radius of 59.4 nm. After catalyst incorporation, the Co@C‐containing composite exhibits a slightly larger characteristic matrix radius of 64.9 nm, whereas the Ni@C‐containing composite shows a reduced size of 52.2 nm. These results suggest that the structural effect of the catalysts depends on the specific catalyst composition, with Ni@C more effectively refining the hydride matrix, while Co@C mainly contributes additional carbon‐scaffold and nanoparticle interfaces without obvious matrix refinement. Meanwhile, the catalyst‐containing composites retain the characteristic distributions of carbon scaffolds and nanoparticles (Figure 5g,h), which are absent in the undoped system. The average radii of the carbon scaffolds are 99.2 and 102.3 nm for Co@C and Ni@C samples, respectively, while the nanoparticle radii remain at 5–6 nm. This demonstrates that the Co@C and Ni@C catalysts are structurally preserved after ball milling and remain embedded as distinct nanoscale/mesoscale components within the hydride matrix. Such a hierarchical arrangement is expected to provide more catalyst–hydride interfacial area than that in the undoped sample.

After five hydrogenation/dehydrogenation cycles, the overall scattering profiles remain distinguishable among the three systems (Figure 5i). The undoped composite shows significant coarsening, with the fitted matrix radius increasing to 129.5 nm (Figure 5j). In contrast, the catalyst‐containing samples show smaller matrix radii (96.6 and 99.1 nm for Co@C and Ni@C, respectively), indicating suppressed agglomeration during cycling. Although slight particle growth is observed in all systems, the increase is less pronounced in the catalyzed samples, demonstrating that the structural refinement is largely preserved. Importantly, the characteristic size distributions of carbon scaffolds and nanoparticles remain largely detectable after cycling (Figure 5k,l), with no evidence of severe coarsening or collapse within the resolution of the model. For example, the fitted carbon scaffold sizes are approximately 170 nm for both catalysts, while the nanoparticle sizes remain within 5–8 nm. The slightly more pronounced growth in the Co@C system is consistent with its comparatively weaker kinetic performance (Figure 2b). These results demonstrate that the addition of Co@C and Ni@C not only introduces catalytic species into the 2LiH + MgB_2_ system, but also restructures the composite across multiple length scales. Compared with the undoped sample, the catalyst‐containing systems exhibit a finer hydride matrix together with stable carbon scaffolds and nanosized particles, leading to a higher density of persistent interfaces. Such structural stabilization provides a plausible basis for the enhanced hydrogen storage kinetics and promotion of the LiBH_4_‐related dehydrogenation step observed in Figures 2, 3, 4.

### 2.6 Electronic Origin of LiBH_4_ Activation

To establish a direct correlation between interfacial electronic interactions and the experimentally observed dehydrogenation behavior, density functional theory (DFT) calculations and in‐situ wide‐angle and small‐angle x‐ray scattering (WAXS & SAXS) techniques were combined (Figure 6). For the Co@C system, the optimized atomic configurations (Figure 6a) reveal that LiBH_4_ strongly interacts with the exposed Co sites, leading to noticeable distortion of the BH_4_^−^ tetrahedra upon relaxation. In contrast to the relatively symmetric configuration in pristine LiBH_4_, these distortions indicate that the local bonding environment of hydrogen is significantly altered by the presence of transition metal sites, suggesting the activation of B─H bonds. Consequently, the calculated dehydrogenation energy barrier of LiBH_4_ is greatly decreased from 110.84 (pristine RHC) to 69.48 kJ mol^−1^ (Figure 6b), confirming that the interfacial interaction effectively facilitates the cleavage of the B─H bond. The corresponding charge density difference (Figure 6c) shows clear electron redistribution at the interface, with electron depletion localized along the B─H bonds and accumulation surrounding the Co atoms. This indicates a strong electronic coupling between the catalyst surface and LiBH_4_, where charge transfer from the hydride species to the metal sites weakens the B─H bond. Therefore, the energy needed for hydrogen release is lowered. In‐situ WAXS & SAXS measurements further capture the system's dynamic evolution, see Figures S2 and S3. The WAXS patterns (Figure 6d) illustrate the gradual transformation of LiBH_4_ during isothermal holding at 400°C, indicating continuous dehydrogenation without any notable emergence of new Co‐containing phases. Meanwhile, the evolution of integrated SAXS intensity and power‐law slope (Figure 6e,f) reflects progressive structural rearrangement at the nanoscale. This suggests that the reaction is accompanied by interface‐mediated morphological changes rather than solely bulk reconstruction. Further evidence of LiBH_4_ consumption and composite rearrangement during dehydrogenation is provided by the continuous decrease in scattering intensity and slope. This implies that hydrogen release is accompanied by continuous reorganization of the catalyst–hydride interface. By limiting severe aggregation and preserving active interfaces throughout cycling, the carbon scaffold facilitates the spatial dispersion of metal sites.

**FIGURE 6:**
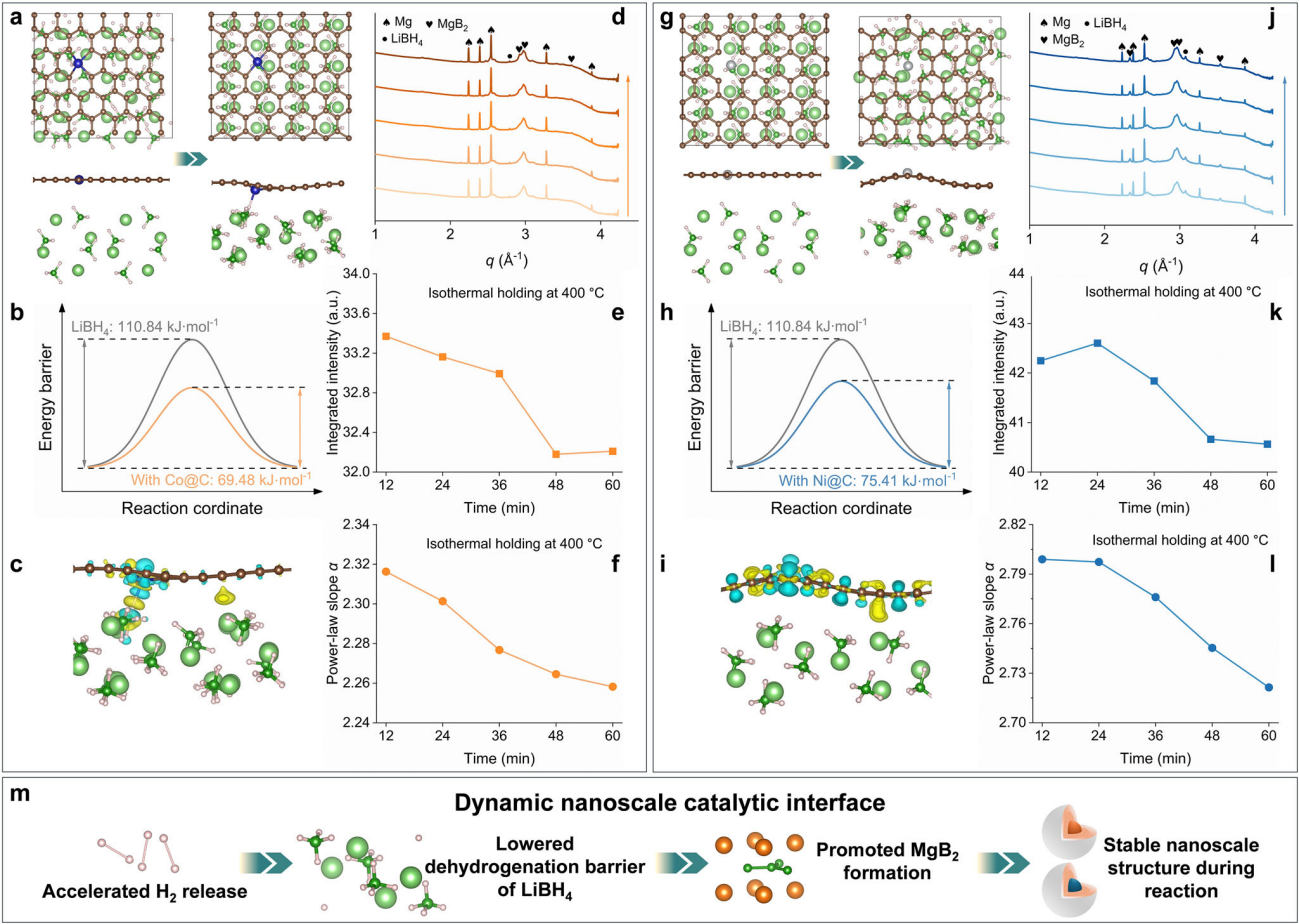
Correlation between interfacial electronic structure and dynamic structural evolution during Co@C/Ni@C catalyzed LiBH_4_ dehydrogenation. (a) Atomic structures of LiBH_4_ and Co@C and their interfacial structures before and after reaction. (b) Calculated dehydrogenation energy barrier for LiBH_4_ interacting with Co@C. (c) Charge density difference at the LiBH_4_ and Co@C interface. (d) In‐situ wide‐angle X‐ray scattering (WAXS) patterns of the hydrogenated 2LiH + MgB_2_ with Co@C during isothermal holding at 400°C. (e), (f) Corresponding evolution of integrated scattering intensity and power‐law slope (*q* = 0.05 to 0.3 Å^−1^) obtained from in‐situ small‐angle x‐ray scattering (SAXS) at various intervals during isothermal holding at 400°C. (g) Atomic structures of LiBH_4_ and Ni@C and their interfacial structures before and after reaction. (h) Calculated dehydrogenation energy barrier for LiBH_4_ interacting with Ni@C. (i) Charge density difference at the LiBH_4_ and Ni@C interface. (j) In‐situ WAXS patterns of the hydrogenated 2LiH + MgB_2_ with Ni@C during isothermal holding at 400°C. (k), (l) Corresponding evolution of integrated scattering intensity and power–law slope (*q* = 0.05 to 0.3 Å^−1^) obtained from in‐situ SAXS at various intervals during isothermal holding at 400°C. (m) Schematic illustration of the interface‐driven catalytic mechanism.

A similar analysis for the Ni@C‐doped system (Figure 6g–i) demonstrates clear interfacial interactions, as evidenced by the distortion of BH_4_^−^ units and charge redistribution at the Ni and LiBH_4_ interface. The calculated dehydrogenation energy barrier (75.41 kJ mol^−1^, Figure 6h) is slightly higher than that of the Co@C‐doped system, indicating a slightly weaker electronic activation at the idealized individual‐interface level. However, this factor is not the only one that determines the overall catalytic performance. Note that the calculated barriers only characterize the intrinsic energetics of idealized local interfaces. Due to its more efficient structural features, such as finer dispersion and a higher density of accessible catalyst–hydride interfaces, the Ni@C‐doped system actually shows faster dehydrogenation kinetics (Figure 2). These structural characteristics may facilitate more extensive interfacial interactions and shorten diffusion pathways, therefore compensating for the slightly higher energy barrier. LiBH_4_ transformation and structural evolution in the Ni@C‐doped sample follow a similar interface‐mediated pathway as the Co@C‐doped system, as confirmed by the in‐situ WAXS & SAXS investigations (Figure 6j–l). Despite the slightly higher calculated energy barrier compared to the Co@C‐doped system, the increased interface density and structural refinement enable a more effective overall dehydrogenation process, which allows for accelerated kinetics. Importantly, the combination of DFT and in‐situ scattering provides an integrated view that links local electronic interactions to the observed reaction dynamics. The catalytic effect on hydrogen release is therefore unlikely to arise solely from a uniform bulk effect. Instead, the catalysts appear to generate electronically active interfaces that interact with LiBH_4_, weakening the B─H bonds, thus lowering the dehydrogenation energy barrier. The associated nanoscale structural evolution is directly captured by the in‐situ WAXS & SAXS measurements, indicating that the reaction proceeds through a continuous interface‐mediated transformation process.

The dramatic improvement in hydrogen storage properties could be mainly attributed to the interfacial interactions between the catalysts and the hydride phases. The reaction in the current system is dominated by reactions taking place at the catalyst–hydride interfaces rather than proceeding uniformly throughout the bulk. The USANS & SANS data (Figure 5) show that the incorporation of Co@C/Ni@C introduces a high density of nanoscale interfaces structurally, which increases the number of active reaction sites. From a kinetic perspective, these interfaces enable shortened diffusion pathways and facilitate hydrogen release, resulting in faster kinetics and lower apparent activation energies (Figures 2 and 3). Both composites with catalysts maintain reduced characteristic matrix radii after five hydrogenation/dehydrogenation cycles compared to the undoped RHC, indicating that the catalyst structures inhibit matrix coarsening. This structural stabilization may be beneficial in preserving short transport distances and fine distribution of the reactants throughout cycling. Furthermore, the populations assigned to the Co or Ni‐containing nanoparticles remain detectable within the 5–8 nm range. The characteristic radius of the Co‐containing nanoparticles after five cycles increase from 5.8 to 7.8 nm, while the radius for Ni‐containing nanoparticles exhibits a minor change from 5.3 to 5.4 nm (Figure 5). The enhanced nanoscale stability of Ni@C may consequently maintain a higher proportion of available catalyst sites. This offers a plausible structural explanation for its superior kinetic performance, even though the calculated intrinsic barrier is higher for the Co‐containing interface. At the atomic scale, the optimized metal‐LiBH_4_ interface models provide a plausible atomistic interpretation of the experimentally observed acceleration of the LiBH_4_^−^related dehydrogenation process. According to the charge‐density‐difference maps, pronounced charge redistribution is localized around the Co or Ni sites and their neighboring BH_4_^−^ units. The asymmetric electron accumulation and depletion suggest electronic coupling at the modeled interfaces, indicating the local electronic environment of BH_4_^−^ units have been modified. These modifications, along with the distortion of the optimized interfacial configurations, are consistent with the enhanced activation of the B─H bonds that are involved in the dehydrogenation process. Consequently, the DFT‐calculated dehydrogenation barrier decreases approximately 37.3% and 32.0% for Co@C and Ni@C‐doped composites, respectively, compared with the pristine RHC. Similar electronic effects have been proposed in previous theoretical and experimental studies, in which interactions between transition‐metal‐containing species and LiBH_4_ alter the local electronic environment of BH_4_^−^ units and thereby promote dehydrogenation [25, 26]. The present computational findings indicate that the modeled catalyst–hydride interface serves as an electronically active region rather than merely a passive physical contact between the catalyst and the hydride. Therefore, the interface simultaneously increases the density of active sites and modulates the intrinsic reactivity of specific hydride species, ultimately governing the overall hydrogen storage performance. Overall, these results indicate that the catalytic mechanism is dominated by an interface‐driven process. Although no crystalline Ni─B compounds are clearly detected in the SR‐PXD/WAXS patterns, the potential formation of nanoscale or amorphous Ni─B interfacial species cannot be completely excluded. Previous research shows that nanosized Ni can react with LiBH_4_ to form Ni_4_B_3_ during dehydrogenation and Ni_2_B after re‐hydrogenation [27]. By modifying the local B─H/B‐related bonding environment, such Ni─B‐related interfacial interactions may contribute to the improved LiBH_4_‐related dehydrogenation kinetics. However, it should be noted that the catalytic role of Ni does not necessarily rely on the formation of detectable crystalline Ni─B compounds. As evidenced in a recent study, Ni clusters can engage strongly with B clusters to form Ni─B heterojunction‐like interfaces with covalent‐like interactions, which promote hydrogen diffusion and weaken B─B bonding [28]. This suggests that Ni─B interfacial/electronic interactions may also modify the local B‐related bonding environment. Therefore, similar interfacial interactions between Co or Ni species and boron‐containing units may take place during LiBH_4_ decomposition in the current system. This catalytic behavior provides a strategy for optimizing hydrogen storage performance in complex hydride systems, in which structural design and electronic regulation are coupled to target specific reaction steps.

## 3 Conclusion

In summary, we demonstrate that the introduction of Co@C/Ni@C catalysts markedly improves the hydrogen storage properties of the 2LiH + MgB_2_ system via interface‐driven processes. By combining both experimental investigations and theoretical calculations, a two‐step dehydrogenation pathway in the RHCs is revealed. LiBH_4_ decomposition constitutes the rate‐limiting step and is significantly facilitated by the catalysts. The incorporation of Co@C/Ni@C not only refines the multi‐scale structure of the composite and preserves stable catalyst–hydride interfaces during cycling, but also promotes strong interfacial electronic interactions that weaken B─H bonds in LiBH_4_. These results suggest that the catalytic enhancement is related to the nanoscale structural characteristics of the Co@C and Ni@C catalysts and involves contributions from catalyst–hydride interfacial interactions. This work provides a basis for further exploring how nanoscale structural characteristics and interfacial electronic regulation can optimize the hydrogen storage properties of materials.

## 4 Experimental Section

*Materials*: Mixing 3.08 g of 2, 4‐ dihydroxybenzoic acid, 0.6 g of ethylenediamine, and 0.934 g of hexamethylenetetramine as organic precursors is the first step for synthesizing the mesoporous carbon. As a block copolymer surfactant template, 3.5 g of Pluronic P123 is dissolved in 80 mL of distilled water. The solution obtained from the first step was transferred into a 120 mL Teflon‐lined stainless‐steel autoclave reactor in the second step, and it was heated at 130°C for 4 h under sealed conditions. The resulting condensed polymer gel in the second step was crushed, repeatedly washed with distilled water, and dried overnight at 50°C in the third step [29]. The final product was found in the form of brown powder (see Figure S4). 5 g of as‐prepared brown powder was mixed with 100 mL diluted NH_4_OH (80 mL distilled water + 20 mL 25% NH_4_OH) using a magnetic stirrer. 0.623 g of Nickel(II) acetate was added, and the mixture was stirred continuously for 6 h at 50°C. Further, the final mixture was filtered and washed with distilled water and ethanol, and dried overnight at 50°C. The pyrolysis process was performed at 600°C for 2 h under an argon atmosphere using a heating rate of 2°C min^−1^. For Co@C synthesis, 2 g of the as‐prepared brown powder was mixed with 100 mL diluted NH_4_OH (80 mL distilled water + 20 mL 25% NH_4_OH) using a magnetic stirrer. 0.280 g of cobalt (II) acetate was mixed, followed by continuous stirring for 6 h at 50°C. The final mixture was filtered and washed with distilled water and ethanol and dried overnight at 50°C. The product was carbonized at 600°C for 2 h under an argon atmosphere using a heating rate of 2°C min^−1^.

Commercially available powders of LiH (95% purity, Sigma–Aldrich) and MgB_2_ (99% purity, Alfa Aesar) were utilized to synthesize 2LiH + MgB_2_ + Co@C/Ni@C composites. 3 g of 2LiH + MgB_2_ with and without 7 wt.% of Co@C/Ni@C were mixed and milled in a Spex 8000 M Mixer Mill for 300 min. Milling was carried out using a stainless‐steel vial and 10 mm diameter stainless steel balls, maintaining a ball‐to‐powder ratio of 20:1. All material handling and milling processes were conducted under Ar atmosphere, with H_2_O and O_2_ levels lower than 1 ppm.

*Hydrogen storage properties*: The hydrogenation and dehydrogenation measurements were conducted using an in‐house‐made Sieverts’ apparatus. The setup includes a reference holder and a sample holder with identical calibrated volumes. The reference holder is empty inside, whereas the sample holder contains approximately 200 mg of the investigated sample. The total calibrated volume of the measurement system was approximately 1024 mL. Both holders were enclosed within a closed electrically heated furnace, and their temperatures were observed using equivalent thermocouples located beside the respective holders. For isothermal measurements, the measurements started after reaching thermal equilibrium at the set temperature. Hydrogen with a purity of 5.0 (99.999%) was used for all the measurements. Both holders were kept at an equivalent temperature and initially charged with hydrogen under identical conditions. Hydrogen uptake/release by the sample induces a pressure difference between the two holders, from which the corresponding hydrogen amount is calculated. The differential–pressure data were recorded with a resolution of 0.01 kPa. For the cycling experiments, the hydrogenation temperature is 350°C and the hydrogen pressure is 50 bar. While the temperature and hydrogen pressure for dehydrogenation are 400°C and 3 bar, respectively. For the in‐situ dehydrogenation measurement, the samples were first hydrogenated at 350°C under 50 bar of hydrogen, then heated from RT to 400°C with a heating rate of 5°C min^−1^ under 3 bar of hydrogen pressure. Note that the measurement uncertainty associated with this technique is approximately 5%. This value is calculated based on hydrogenation/dehydrogenation measurements carried out on a broad range of temperatures and pressures for a standard sample of MgH_2_. To calculate the *E*_a_ for the hydrogenation process, the samples were hydrogenated at various temperatures under 50 bar of hydrogen. To calculate the dehydrogenation *E*_a_, the samples were first hydrogenated at 350°C under 50 bar of hydrogen, then dehydrogenated under 3 bar of hydrogen pressure at different temperatures. The reaction fraction (*α*) was determined from the normalized hydrogen uptake/release. The rate constant (*k*) was derived by fitting the isothermal kinetic curves using the Johnson–Mehl–Avrami–Kolmogorov (JMAK) model over an *α* range of 0.05 to 0.95. The JMAK model is expressed in the following equation [30]:

(2) $$\alpha \left(t\right)=1-e^{-kt^{n}}$$

where *t* is the reaction time and *n* is the Avrami exponent. The identical fitting approach was applied to all samples to ensure consistent comparison. The calculations of *E*_a_ are based on the Arrhenius equation [31, 32]:

(3) $$k=A\cdot e^{\frac{-E_{a}}{RT}}\cdot \mathrm{In}\frac{p}{p_{\mathrm{eq}}}$$

where *A* is the pre‐exponential factor, R is the gas constant, *T* is the absolute temperature, *p* is the hydrogen pressure and *p*_eq_ is the equilibrium pressure. Then *E*_a_ can be determined by the Arrhenius plot fitting of ln*k* versus *T* ^−1^.

*Characterizations*: The morphology, structural evolution, thermal behavior and crystallographic properties of the synthesized nanocomposites Co@C and Ni@C have been carefully examined by SEM, TEM, STA–DTA and XRD investigations. The surface morphology of the synthesized Co@C and Ni@C nanocomposites was examined using a Quanta 3D FEG scanning electron microscope at the Department of Inorganic and Applied Chemistry, University of Hamburg, Germany. The morphology and distribution of Co and Ni nanoparticles within the carbon framework were studied using a JEM‐1011 transmission electron microscope at the Department of Inorganic and Applied Chemistry, University of Hamburg, Germany. Thermal characteristics and stability of the prepared Co@C and Ni@C nanocomposites were investigated by an STA 449 F3 Jupiter (NETZSCH‐Gerätebau GmbH, Germany) equipped with a SiC furnace and a Type S TGA–DTA sensor. The crystal structure and phase composition of the prepared Co@C and Ni@C nanocomposites were determined by x‐ray diffractometer X'Pert Pro PW3040/60 (PANalytical) in *θ*/*θ* (Bragg–Brentano) geometry with Cu(*K*α) radiation (45 kV, 40 mA), counting time of 74 s, and step size of 0.013° (2*θ*).

The phase constitutions of the as‐milled and cycled 2LiH + MgB_2_ + Co@C/Ni@C composites were analyzed by SR‐PXD at beamline P02.1 at PETRA III of DESY (Hamburg, Germany). The samples were loaded into borosilicate glass capillaries and sealed under Ar atmosphere using UV glue. While the in‐situ dehydrogenation measurements of 2LiH + MgB_2_ + Co@C/Ni@C composites were performed from RT to 425°C with a heating rate of 5°C min^−1^ under 3 bar of hydrogen pressure, using a custom‐designed high‐pressure cell [33]. A wavelength of 0.20739 Å was used, and diffraction patterns were recorded with a Varex 4343CT detector. The sample‐to‐detector distance (SDD) was calibrated using a CeO_2_ standard. The collected 2D images were subsequently integrated into numerical data using the pyFAI program [34].

USANS & SANS experiments were conducted at the Australian Centre for Neutron Scattering, ANSTO (Lucas Heights, Australia), using the Kookaburra (USANS) instrument and the Quokka (SANS) instrument, respectively [35, 36]. Kookaburra uses a highly oriented pyrolytic graphite pre‐monochromator to select neutrons, which are further refined by five boused silicon 111 or silicon 311 to select wavelengths of 4.74 and 2.37 Å. A wavelength of 4.74 Å was selected for the USANS measurements in order to provide sufficient scattering overlap with the Quokka SANS data. The combined measurements covered a *q* range of approximately 4 × 10^−5^ to 8 × 10^−1^ Å^−1^. SANS measurements were performed at sample‐to‐detector distances of 1.3, 12 and 20 m, with MgF_2_ lenses used for the 20 m configuration. Incident neutron wavelengths of 5 Å was used at 1.3 and 12 m, and 8.1 Å was used at 20 m. Absolute intensity calibration was performed using a direct beam transmission measurement. Furthermore, measurements of the direct (empty) beam, blocked beam, and an empty sample holder were conducted to facilitate accurate background subtraction and transmission corrections. The 1D USANS data were reduced and desmeared using standard procedures described in the literature [37]. SANS data from the three configurations were reduced and merged using the standard Quokka data‐reduction macros implemented in Igor Pro [38]. Subsequently, the SANS and desmeared USANS data were merged and fitted using SASfit software package [39].

In‐situ WAXS & SAXS measurements of 2LiH + MgB_2_ + Co@C/Ni@C composites were performed at the SAXSMAT beamline 62 at PETRA III [40]. The samples were heated up from RT to 400°C with a heating rate of 10°C min^−1^ under 3 bar of hydrogen pressure, using a custom‐designed high‐pressure cell [33]. The measurements were performed at an average SDD of 6 m using an incident photon energy of 12 keV, corresponding to a wavelength of *λ* = 0.103 nm. The data were acquired at an acquisition rate of 1 s per frame, using an Eiger 2×9 M area detector for acquisition of in‐situ SAXS data, while WAXS data were recorded simultaneously using an Eiger 2×4 M area detector. The *q*‐axis calibration for the SAXS measurements was achieved using a silver behenate standard, whereas the *q*‐axis for the WAXS data were calibrated using a LaB_6_ standard. The resulting datasets were subsequently time‐binned and averaged to improve data quality and reduce statistical errors.

*Computational method*: DFT implementation in CP2K/Quickstep was based on a hybrid Gaussian plane wave (GPW) scheme [41]. An atom‐centered Gaussian‐type basis set represented the orbitals, and an auxiliary plane wave basis set was utilized to re‐expand the electron density in the reciprocal space. The Perdew–Burke–Ernzerhof (PBE) function was employed to describe the exchange‐correlation function, with all calculations incorporating Grimme D3 (DFT‐D3) dispersion correction [42, 43]. The calculations used Goedecker–Teter–Hutter (GTH) pseudopotentials and the Gaussian basis set was double‐zeta with one set of polarization functions (DZVP) [44, 45, 46].

The wave function was optimized by a matrix diagonalization process in the structural optimization. Furthermore, geometrical configurations were optimized using the Broyden–Fletcher–Goldfarb–Shanno (BFGS) minimizer in calculations with a (5 × 5 × 5) Monkhorst‐Pack *k*‐point grid and plane wave cutoff energy of 400 Ry. Subsequently, an optimized heterojunction structure comprising LiBH_4_ and Co@C/Ni@C is constructed, with a 15 Å vacuum layer incorporated in the Z direction to mitigate periodic effects.

Molecular dynamics (AIMD) simulations were employed and canonical ensemble conditions were imposed by the canonical sampling through velocity rescaling thermostat. The MD time step was set to 1fs. For all the MD trajectories, the initial simulation results of 2 ps (2000 steps) were abandoned to avoid the effect of non‐equilibrium, and then followed results of 8 ps (8000 steps) were selected for dehydrogenation simulation. Besides, all MD calculations used orbital transformation algorithm and the *Γ* point to relax structures after expansion.

The post‐processing analysis of MD used the visualization program VMD to calculate the radial distribution functions (RDFs) of the B─H bonds [47, 48]. The potential of mean force (PMF) between B─H bonds can be obtained by further processing the RDF of B─H bonds using Equation (4). The PMF can be calculated as:

(4) $$\Delta G_{a}\left(r\right)=k_{B}T\;\mathrm{In}\frac{g_{\max}\left(r\right)}{g_{\min}\left(r\right)}$$

where *k*_B_ is Boltzmann's constant, *T* is the environmental temperature, but this is always 1100 K in our calculations, ∆*G*_a_(*r*) is the dissociation energy of B─H bonds, *g*_max_(*r*) and *g*_min_(*r*) is the maximum and minimum values of RDF. The electron density difference was calculated to analyze the electron transfer between LiBH_4_ and Co@C/Ni@C.

## Author Contributions

**Yuanyuan Shang**: conceptualization, methodology, data curation, investigation, validation, visualization, and writing – original draft. **Pawan Kumar Soni**: methodology, investigation, and writing – review and editing. **Han Ge**: software, formal analysis, and writing – review and editing. **Weijie Yang**: software, resources, and writing – review and editing. **Fahim Karimi**: investigation, formal analysis, and writing – review and editing. **Thi Thu Le**: investigation, formal analysis, and writing – review and editing. **Tamás Simon**: investigation, formal analysis, and writing – review and editing. **Michael Fröba**: funding acquisition, project administration, resources, and supervision. **Jitendra Mata**: resources, formal analysis, and writing – review and editing. **Joshua King**: resources, formal analysis. **Hai‐Wen Li**: formal analysis, writing – review and editing. **Thomas Klassen**: funding acquisition, project administration, resources, and supervision. **Claudio Pistidda**: conceptualization, project administration, funding acquisition, supervision, resources, and writing – review and editing.

## Funding

This research was funded by the Bundesministerium für Forschung, Technologie und Raumfahrt (BMFTR) under the project of HyPoKo (grant number 03SF0657A‐D). Additional funding was received from dtec.bw – Digitalization and Technology Research Center of Bundeswehr which we gratefully acknowledge. Fahim Karimi would like to acknowledge the financial support provided by the project Digitale Material Manufaktur (DigiMatMan), funded by dtec.bw – Digitalization and Technology Research Center of the Bundeswehr. The dtec.bw initiative is financed by the European Union – NextGenerationEU.

## Conflicts of Interest

The authors declare no conflicts of interest.

## Supporting information




**Supporting File**: adma75169‐sup‐0001‐SuppMat.docx.

## Data Availability

The data that support the findings of this study are available from the corresponding author upon reasonable request.
