# Supplementary material for: Interface‐Mediated Catalysis for Improved Hydrogen Storage in Reactive Hydride Composites
Source: Adv Mater. 2026 Sep 25;38(63):e75169. doi: 10.1002/adma.75169 (PMC13755663; doi:10.1002/adma.75169)
Supplement: Supplementary file 1 [file ADMA-38-e75169-s001.docx]

Supporting Information

Interface-mediated catalysis for improved hydrogen storage in reactive hydride composites

Yuanyuan Shang*, Pawan Kumar Soni, Han Ge, Weijie Yang, Fahim Karimi, Thi Thu Le, Tamás Simon, Michael Fröba, Jitendra Mata, Joshua King, Hai-Wen Li, Thomas Klassen, Claudio Pistidda*

Yuanyuan Shang, Fahim Karimi, Thi Thu Le, Thomas Klassen, Claudio Pistidda

Materials Design, Institute of Hydrogen Technology, Helmholtz-Zentrum Hereon, 21502 Geesthacht, Germany
E-mail: [yuanyuan.shang@hereon.de](mailto:yuanyuan.shang@hereon.de), [claudio.pistidda@hereon.de](mailto:claudio.pistidda@hereon.de)

Pawan Kumar Soni
Department of Physics, School of Sciences, IILM University, Greater Noida, India

Pawan Kumar Soni, Tamás Simon, Michael Fröba

Institute of Inorganic and Applied Chemistry, University of Hamburg, Martin-Luther-King-Platz 6, 20146 Hamburg, Germany

Han Ge

Jinshuitan Hydropower Plant of State Grid Zhejiang Electric Power Co., Ltd, China

Han Ge, Weijie Yang

School of Energy and Power Engineering, North China Electric Power University, Baoding, 071003 Hebei, China

Michael Fröba

The Hamburg Centre for Ultrafast Imaging, Luruper Chaussee 149, 22761 Hamburg, Germany

Jitendra Mata, Joshua King

Australian Centre for Neutron Scattering, ANSTO, New Illawarra Rd, Lucas Heights, 2234, NSW, Australia

Jitendra Mata

School of Chemistry, University of New South Wales, Sydney 2052, Australia

Jitendra Mata

Faculty of Science & Technology, Charles Darwin University (CDU), Darwin, NT 0810, Australia

Hai-Wen Li

School of Advanced Energy, Sun Yat-sen University, 518107 Shenzhen, China

Thomas Klassen

Helmut Schmidt University, Holstenhofweg 85, 22043 Hamburg, Germany

Figure

**Figure S1. Hydrogenation/dehydrogenation cycling curves of 2LiH + MgB_2_ + Co@C/Ni@C composites. a** The overall hydrogenation/dehydrogenation kinetics of the undoped 2LiH + MgB_2_ composites over 5 cycles. **b** The overall hydrogenation/dehydrogenation kinetics of the Co@C-doped 2LiH + MgB_2_ composites over 5 cycles. **c** The overall hydrogenation/dehydrogenation kinetics of the Ni@C-doped 2LiH + MgB_2_ composites over 5 cycles.

**Figure S2. *In-situ* WAXS & SAXS patterns of the hydrogenated 2LiH + MgB_2_ with Co@C during dehydrogenation from RT to 400 ºC (heating rate of 10 C min^-1^), followed by isothermal holding at 400 ºC. a** WAXS patterns. **b** Corresponding SAXS patterns.

**Figure S3. *In-situ* WAXS & SAXS patterns of the hydrogenated 2LiH + MgB_2_ with Ni@C during dehydrogenation from RT to 400 ºC (heating rate of 10 C min^-1^), followed by isothermal holding at 400 ºC. a** WAXS patterns. **b** Corresponding SAXS patterns.

**Figure S4. Optical image of crushed condensed polymer gel after hydrothermal treatment.**

Table

**Table S1. Fitting parameters obtained from the combined sphere-model analysis of the USANS & SANS data.**

| **Sample** | **Sphere population** | **Mean *r* (nm)** | **Lognormal width** | **Polydispersity** | **Scaling** | **Volume-based relative fraction** |
| --- | --- | --- | --- | --- | --- | --- |
| Co@C | Nanoparticle | 6.64 | 0.3787 | 39.27% | 1.4189 × 10^-11^ | 38.95% |
|  | Carbon scaffold | 155.65 | 0.6220 | 69.53% | 5.2250 × 10^-15^ | 61.05% |
| Ni@C | Nanoparticle | 5.78 | 0.4176 | 43.65% | 5.0005 × 10^-12^ | 47.14% |
|  | Carbon scaffold | 170.57 | 0.6391 | 71.57% | 6.3648 × 10^-16^ | 52.86% |
| As-milled 2LiH + MgB_2_ | 2LiH + MgB_2_ matrix | 59.38 | 0.6002 | 65.85% | 8.0609 × 10^-14^ | 100% |
| As-milled 2LiH + MgB_2_ + Co@C | 2LiH + MgB_2_ matrix | 64.89 | 0.6359 | 70.59% | 4.4739 × 10^-14^ | 99.57% |
|  | Nanoparticle | 5.83 | 0.2338 | 23.71% | 2.2237 × 10^-14^ | 0.17% |
|  | Carbon scaffold | 99.23 | 0.4777 | 50.63% | 1.4418 × 10^-17^ | 0.26% |
| As-milled 2LiH + MgB_2_ + Ni@C | 2LiH + MgB_2_ matrix | 52.23 | 0.6057 | 66.57% | 8.3917 × 10^-14^ | 98.07% |
|  | Nanoparticle | 5.29 | 0.2326 | 23.57% | 2.9598 × 10^-14^ | 0.15% |
|  | Carbon scaffold | 102.30 | 0.4743 | 50.23% | 1.0670 × 10^-16^ | 1.78% |
| Cycled 2LiH + MgB_2_ | 2LiH + MgB_2_ matrix | 129.46 | 0.5759 | 62.71% | 3.9059 × 10^-16^ | 100% |
| Cycled 2LiH + MgB_2_ + Co@C | 2LiH + MgB_2_ matrix | 96.58 | 0.63355 | 70.28% | 4.7738 × 10^-15^ | 86.74% |
|  | Nanoparticle | 7.82 | 0.4327 | 45.38% | 4.2266 × 10^-14^ | 1.21% |
|  | Carbon scaffold | 170.03 | 0.6379 | 70.87% | 1.8983 × 10^-17^ | 2.05% |
| Cycled 2LiH + MgB_2_ + Ni@C | 2LiH + MgB_2_ matrix | 99.07 | 0.6336 | 70.28% | 4.0496 × 10^-15^ | 95.75% |
|  | Nanoparticle | 5.41 | 0.3934 | 40.91% | 1.4547 × 10^-13^ | 1.72% |
|  | Carbon scaffold | 174.55 | 0.6421 | 71.43% | 2.0544 × 10^-17^ | 2.53% |
